# Two novel CMY-2-type β-lactamases encountered in clinical *Escherichia coli* isolates

**DOI:** 10.1186/s12941-015-0070-8

**Published:** 2015-03-18

**Authors:** Vera Manageiro, Eugénia Ferreira, Margarida Pinto, Fernando Fonseca, Mónica Ferreira, Richard Bonnet, Manuela Caniça

**Affiliations:** Department of Infectious Diseases, National Reference Laboratory of Antibiotic Resistances and Healthcare Associated Infections, National Institute of Health Dr. Ricardo Jorge, Av. Padre Cruz, 1649-016 Lisbon, Portugal; Centre for the Study of Animal Sciences (ICETA), University of Oporto, Oporto, Portugal; Laboratory of Microbiology, Hospital Garcia de Orta, EPE, Almada, Portugal; Laboratory of Clinical Pathology, Hospital de Santa Luzia, Viana do Castelo, Portugal; CHU Clermont-Ferrand, Laboratoire de Bactériologie, Clermont-Ferrand, France; Present address: Laboratory of Microbiology, Centro Hospitalar de Lisboa Central, EPE, Lisbon, Portugal; Present address: Laboratory of Clinical Pathology, Centro Hospitalar de Póvoa de Varzim-Vila do Conde, EPE, Póvoa de Varzim, Portugal

**Keywords:** β-lactamase, Resistance regions, Genetic environment, *Escherichia coli*

## Abstract

**Background:**

Chromosomally encoded AmpC β-lactamases may be acquired by transmissible plasmids which consequently can disseminate into bacteria lacking or poorly expressing a chromosomal *bla*_AmpC_ gene. Nowadays, these plasmid-mediated AmpC β-lactamases are found in different bacterial species, namely *Enterobacteriaceae*, which typically do not express these types of β-lactamase such as *Klebsiella* spp. or *Escherichia coli*. This study was performed to characterize two *E. coli* isolates collected in two different Portuguese hospitals, both carrying a novel CMY-2-type β-lactamase-encoding gene.

**Findings:**

Both isolates, INSRA1169 and INSRA3413, and their respective transformants, were non-susceptible to amoxicillin, amoxicillin plus clavulanic acid, cephalothin, cefoxitin, ceftazidime and cefotaxime, but susceptible to cefepime and imipenem, and presented evidence of synergy between cloxacilin and cefoxitin and/or ceftazidime. The genetic characterization of both isolates revealed the presence of *bla*_CMY-46_ and *bla*_CMY-50_ genes, respectively, and the following three resistance-encoding regions: a *Citrobacter freundii* chromosome-type structure encompassing a *blc-sugE-bla*_CMY-2-type_*-ampR* platform; a *sul*1-type class 1 integron with two antibiotic resistance gene cassettes (*dfrA1* and *aadA1*); and a truncated mercury resistance operon.

**Conclusions:**

This study describes two new *bla*_CMY-2-type_ genes in *E. coli* isolates, located within a *C. freundii*-derived fragment, which may suggest their mobilization through mobile genetic elements. The presence of the three different resistance regions in these isolates, with diverse genetic determinants of resistance and mobile elements, may further contribute to the emergence and spread of these genes, both at a chromosomal or/and plasmid level.

## Background

AmpC β-lactamases, along with Class A β-lactamases, are a major group of clinically important enzymes [1,2]. They belong to class C according to the Ambler classification and to group 1 following the functional classification of Bush-Jacoby [3,4], whose prevalence is increasing worldwide [1]; these β-lactamases are associated with infections caused by pathogenic Gram-negative bacteria, particularly *Escherichia coli* and *Klebsiella pneumoniae*. The identification of isolates containing plasmid-mediated AmpC-β-lactamase (PMAβ) is epidemiologically and clinically relevant due to the limitations of treatment options [5].

AmpC enzymes hydrolyse amino- and ureidopenicillins, and cephamycins (cefoxitin and cefotetan) and, at a low level, oxyiminocephalosporins (ceftazidime, cefotaxime, and ceftriaxone) and aztreonam: they are not inhibited by β-lactamase inhibitors such as clavulanic acid [1]. AmpC-producing isolates are susceptible to carbapenems and to zwitterionic cephalosporins (cefepime and cefpirome).

In this study, we performed the phenotypic and molecular characterization of two new CMY-2-types (designated CMY-46 and CMY-50), both encoded by probably chromosomal inducible *ampC* genes, produced by two clinical *E. coli* isolates. The genetic environment of *bla*_CMY-46_ and *bla*_CMY-50_ was also investigated.

## Methods

### Bacterial isolate collection

Two clinical *E. coli* strains (INSRA1169 and INSRA3413) were isolated, in 1999, from urine samples of two patients of 77 years and 7 months old, in two different hospitals in Portugal. *E. coli* DH5α (pBK-CMY-2) strain was used as control for antimicrobial susceptibility tests.

### Antimicrobial susceptibility tests

Minimal inhibitory concentrations were determined by a microdilution method according to guidelines of the French Society of Microbiology (SFM 2013, http://www.sfm-microbiologie.org/) against seven β-lactams, alone or in combination with clavulanic acid, and against ciprofloxacin, gentamicin and trimethoprim. Isolates non-susceptible to one third-generation cephalosporin, cefoxitin and/or exhibiting synergy with boronic acid and/or cloxacillin, were considered as presumptive AmpC producers. Imipinem and clavulanic acid were used in order to identify induction effect of AmpC [1,6]. Disks of inducing agents (imipenem 10 μg and amoxicillin plus clavulanic acid 25 + 10 μg) and disks of cephalosporins (cefotaxime 30 μg and ceftazidime 30 μg) were placed on Mueller–Hinton agar plates, 20 mm apart. Positive induction was demonstrated by the antagonism effect surrounding the cephalosporin disks adjacent to the inducers.

### Isoelectric point determination

β-Lactamases were characterized by isoelectric focusing of ultrasonicated bacterial extracts with the control strains expressing pI 5.2, 5.6, 7.6, 9.0, 9.2, as previously described [7].

### Molecular characterization of *ampC* and ESBL-encoding genes

The presence of acquired *ampC* (*bla*_CMY_, *bla*_MOX_, *bla*_FOX_, *bla*_LAT_, *bla*_ACT_, *bla*_MIR_, *bla*_DHA_, *bla*_MOR_, *bla*_ACC_) and *bla*_ESBL_ genes (*bla*_TEM_, *bla*_SHV_, *bla*_OXA-1-type_, *bla*_CTX-M_) was investigated by multiplex PCR assays with primers and conditions as described elsewhere [7-10], and those from Table 1. Controls were included in all assays.

**Table 1:** **Primers, drawn in this study, used for PCR amplification and sequencing of PMAβ genes and for PCR mapping of** ***bla***_**CMY-46**_ **and** ***bla***_**CMY-50**_

***Gene (s)***	***Primer Sequence (5’ → 3’)***		**PCR product (bp) / Method** ^**a**^
	***Forward***	***Reverse***	
*bla*_CMY-G2_	TTACGGAACTGATTTCATG	TCGTCAGTTATTGCAGC	1169 / PCR + Seq.
*orf513*	GCCAGGTCTTGAGTATCGTC	CATGTAATTGAGTCAGCGTATC	363 / PCR + Seq.
*fdrB - fdrD*	CTCAGTTGACCACCACGAAC	GAATGCCAATAGCCGTTACGAC	920 / PCR + Seq.
*fdrB - ampR*	CTCAGTTGACCACCACGAAC	CACCAGTCAGAATGTTCACGCA	1140 / PCR + Seq.
*ampR - bla*_*CMY-G2*_	TGCGTGAACATTCTGACTGGTG	TTTCTCCTGAACGTGGCTGGC	1660 / PCR + Seq.
*bla*_*CMY-G2*_ *- sugE*	TGGCCAGAACTGACAGGCAAA	ATGTCCTGGATCGTTTTATTA	1751 / PCR + Seq.
*merA- urf2*	TTCCCCTACCTGACGATGG	TGTTGCAGGCAGGAATAGC	1214/ PCR + Seq.
*merR - merA*	TCTTTCTCCCCTTGCAGCG	CACCTTGTCGAACAGCCCA	Variable / PCR + Seq.
*merA*	CGTCCAATCTGCCATAGTG	GTAGGGGAACAACTGGTCG	Seq.
*merD*	CCTTCGAGGCGGGTATC	CCGATACCCGCCTCGAAG	Seq.

^a^Method used for screening and/or identification of genes: M-PCR, Multiplex-PCR; Seq, sequencing; PCR + Seq, PCR and sequencing.

### Gene transfer experiments

Transferability of the *bla*_CMY_ genes was attempted by both broth mating-out assays and electroporation. Conjugation experiments were performed at 37°C, using recipient strains *E. coli* C600 Rif^R^, Str^R^ and *E. coli* J53 NaN_3_^R^, according to the antibiotic susceptibilities of the clinical isolates used as donor. Transconjugants were selected on MacConkey agar plates containing 250 μg/ml of rifampicin, 160 μg/ml of streptomycin or 160 μg/ml of sodium azide plus 10 μg/ml of cefoxitin. Plasmid DNA was extracted from clinical strains, using the Wizard Plus Midipreps DNA Purification kit (Promega), and used to transform electrocompetent *E. coli* DH5α ∆*ampC* by electroporation, as previously described [7]. Transformants were selected on Luria broth medium containing 10 μg/ml of cefoxitin.

### Cloning experiments

The *bla*_CMY-2_, *bla*_CMY-46_ and *bla*_CMY-50_ genes were amplified with iProof^TM^ High-Fidelity DNA Polymerase (Bio-Rad, Hercules, CA), using primers from Table 1. Amplicons (1169 bp) were ligated in the *Sma*I site of the phagemid pBK-CMV (Stratagene) downstream of its inducible *lacZ* promoter and transformed into electrocompetent *E. coli* DH5α ∆*ampC* cells. A gene Pulser II apparatus (Bio-Rad, Hercules, CA) was used for standard electroporation techniques, as previously described [7]. Recombinant bacteria were selected on LB agar plates containing 10 μg/ml of cefoxitin.

### Genetic background characterization

The presence of class 1 integrons was determined in both isolates through PCR amplification of the integrase-specific *intI1* gene with the same specific primers and conditions as reported previously [11] (Table 1). PCR-mapping and sequencing of the genetic environment of *bla*_CMY-46_ and *bla*_CMY-50_ was performed using primers targeting genes known for promoting antibiotic resistance and integrons (Table 1). Sequence alignments and generation of resistance cassette contigs were performed using *Bionumerics* (Applied Maths). Gene identity was confirmed at the NCBI website (http://www.ncbi.nlm.nih.gov/).

## Findings

The two clinical *E. coli* isolates INSRA1169 and INSRA3413 were resistant to amoxicillin, amoxicillin plus clavulanic acid, cephalothin, cefoxitin, ceftazidime, cefotaxime, gentamicin and trimethoprim, but susceptible to cefepime and imipenem (Table 2). INSRA1169 was also nonsusceptible to ciprofloxacin. Synergy between cloxacillin and cefoxitin plus cefotaxime and boronic acid, along with the absence of synergy between extended-spectrum cephalosporins and clavulanic acid, suggest that the resistance to extended-spectrum cephalosporins was mediated by the overproduction of AmpC β-lactamases. The resistance phenotype was not transferable, neither in conjugation assays with *E. coli* C600 as a recipient, or in transformation assays by electroporation of plasmid-DNA preparations into *E. coli* DH5α. This might suggest a chromosomal location of AmpC-encoding genes.

**Table 2:** **MICs of antibiotics for CMY-46- and CMY-50-producing** ***E. coli*** **isolates and** ***E. coli*** **transformants and recipients** ^***a***^

***E. coli*** **strain**	**MIC (μg/ml)** ^**b**^											
	**AMX**	**AMC** ^**c**^	**CF**	**CAZ**	**CCAZ** ^**c**^	**CTX**	**FEP**	**FOX**	**IMP**	**CIP**	**GEN**	**TMP**
DH5α Δ*ampC*	8	8	8	0.25	0.125	0.06	0.03	4	0.25	≤0.125	≤0.125	≤0.25
DH5α (pBK-CMY-2)	>2048	>2048	1024	32	16	8	0.25	64	0.5	≤0.125	1	≤0.25
INSRA1169 (CMY-46 + TEM-1)	>2048	>2048	1024	16	4	4	0.032	64	2	4	>128	>128
DH5α (pBK-CMY-46)	>2048	>2048	1024	32	4	8	0.25	64	0.5	≤0.125	2	≤0.25
INSRA3413 (CMY-50)	>2048	>2048	1024	16	8	4	0.25	64	2	≤0.125	64	64
DH5α (pBK-CMY-50)	>2048	>2048	1024	16	8	4	0.25	64	0.5	≤0.125	0.5	≤0.25

^a^ *E. coli* EcDH5α (pBK-CMY-2) was control strain; *E. coli* DH5α (pBK-CMY-46) and *E. coli* DH5α (pBK-CMY-50) were transformants of *E. coli* INSRA1169 (harboring CMY-46 and TEM-1 enzymes) and *E. coli* INSRA3413 (harboring CMY-50 enzyme), respectively; *E. coli* EcDH5α was the recipient strain.

^b^AMX, amoxicillin; AMC, amoxicillin-clavulanic acid; CF, cephalothin; CAZ, ceftazidime; CCAZ, ceftazidime-clavulanic acid; CTX, cefotaxime; FEP, cefepime; FOX, cefoxitin; IMP, imipenem; CIP, ciprofloxacin; GEN, gentamicin and TMP, trimethoprim.

^c^Clavulanic acid, at fixed concentration of 2 μg/ml.

**Figure 1:**
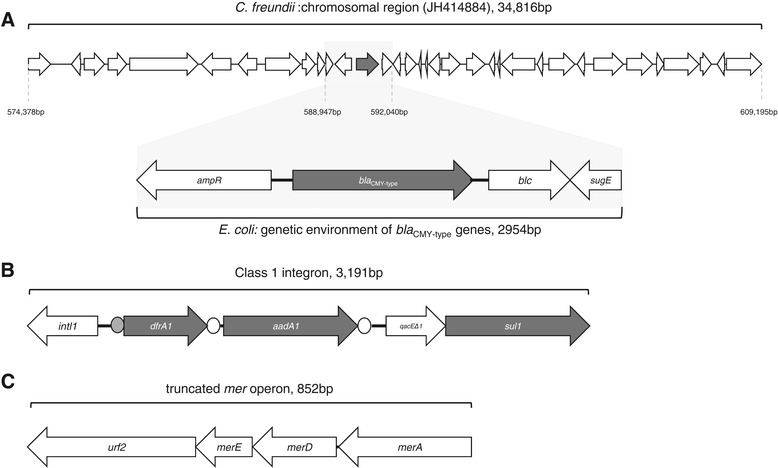
**Schematic representation of the same three structures found within clinical isolates expressing** ***bla***_**CMY-46**_ **and** ***bla***_**CMY-50**_ **.** The directions of transcription of the corresponding genes are depicted by arrows. **A**: sequence, including the genetic environment of *bla*_CMY-type_ genes, compared with *C. freundii* chromosomal region (GenBank JH414884); **B**: class 1 integron, with *attI1* site (grey circle) and the two *attc* regions (open circles); **C**: truncated mercury resistance operon.

Transformants, obtained after cloning of DNA amplicons of INSRA1169 and INSRA3413 in pBK-CMV plasmid vector (Figure 1a), showed a resistance phenotype similar to that of clinical isolates. However, they were susceptible to ciprofloxacin, gentamicin and trimethoprim, like the control strain *E. coli* DH5α (pBK-CMY-2) (Table 2). Both the clinical strains and the transformants produced β-lactamases exhibiting an alkaline isoelectric point (pI 9.2) compatible with AmpC-type β-lactamases. Indeed, PCR revealed the absence of *bla*_ESBL_ genes plus the presence of chromosomal *E. coli ampC*-type gene; the sequence of cloned DNA fragments identified two new CMY-2-like genes, which were not related to chromosome-mediated *E. coli* AmpC gene. The chromosomal location of such genes has only been observed in *Salmonella* spp. and *Proteus mirabilis* isolates [12-14].

The deduced amino acid sequences confirmed that the new genes encoding the β-lactamases CMY-46 (in INSRA1169) and CMY-50 (in INSRA3413), which were new variants of CMY-2, differed by 9 and 13 amino acid substitutions, respectively (Table 3). Two of these mutations (Q193K plus P208A for CMY-46 and N194S plus D198N for CMY-50) are in the Ω loop (between amino acids 178 and 226), which interacts by hydrogen bonding with helix H-2 close to the active Ser64. Substitutions in this region have been linked to the extension of the hydrolysis spectrum [15]. However, CMY-46 and CMY-50 β-lactamases did not confer resistance to cefepime and conferred low level of resistance to ceftazidime and cefotaxime, which suggests that, in contrast to extended-spectrum AmpCs, they have moderate or no extended-spectrum activity (Table 2) [15-18]*.*

**Table 3:** **Comparison of amino acid substitutions of two new CMY-type β-lactamases**

**PMAβ**	**Amino acid at position no.** ^**a**^																				**pI**	**Accession** **Number**
	**3**	**3**	**4**	**1**	**1**	**1**	**1**	**1**	**1**	**1**	**1**	**1**	**1**	**1**	**2**	**2**	**2**	**2**	**2**	**3**		
	**2**	**5**	**9**	**0**	**0**	**2**	**2**	**3**	**4**	**6**	**9**	**9**	**9**	**9**	**0**	**3**	**4**	**5**	**6**	**4**		
				**2**	**5**	**4**	**6**	**3**	**3**	**4**	**0**	**3**	**4**	**8**	**8**	**6**	**2**	**3**	**1**	**8**		
CMY-2	V	Q	A	Q	R	D	R	H	T	K	T	Q	N	D	P	A	H	A	R	V	9.0	X91840
CMY-46			T		S		T	R	A			K			A	V	R				9.2	FN556186
CMY-50	I	E		R	S	E	T	R		Q			S	N				E	C	A	9.2	FN645444

^a^Numbering according to Bauernfeind *et al*., 1996 [19].

The study of sequences surrounding *bla*_CMY-46_ and *bla*_CMY-50_ revealed the presence of the *blc* gene (encoding an outer membrane lipoprotein) and the *sugE* gene (encoding a small MDR protein responsible for resistance to quaternary ammonium compounds) downstream of their open reading frames (Figure 1a). Upstream, an *ampR* gene encoding the usual transcriptional regulator of *ampC* genes was observed in an opposite direction of transcription (Figure 1a). The presence of an intact *ampC*-*ampR* segment in both new *bla*_CMY_ genetic regions implied that the production of CMY-46 and CMY-50 is inducible, which was corroborated by the used phenotypic induction method. This *ampC*-*ampR* region was identical to the sequence flanking the *bla*_AmpC_ gene in the *C. freundii* chromosome [6], except for AmpR_CMY-46_ (that had 4 amino acid substitutions), but none were located in the helix-turn-helix region or in other positions related to AmpR function [20,21]. The promoter regions of our *bla*_CMY-2-type_ and *ampR* genes harbored no sequence element associated with increased strength of the promoter [20,21]. In addition, the *frdD*, *frdC*, and *frdB* genes that are usually adjacent to *ampC-ampR* in the *C. freundii* chromosome were not identified in the sequences flanking *bla*_CMY-46_ or *bla*_CMY-50_.

Class 1 integrons, also detected in INSRA1169 and INSRA3413 (Figure 1b), comprised the integrase-encoding gene *intI1*, two gene cassettes, *aacA1* and *dfrA1*, and *qacEΔ1* plus *sul1*, which were probably responsible for the observed resistances to trimethoprim and aminoglycosides. We also found a truncated mercury resistance operon (Figure 1c), which was previously reported as belonging to a “*kan* region” that included a kanamycin resistance gene [22]. This finding is of concern since mercury resistance may help to promote antibiotic resistance through indirect selection [23].

In summary, this study describes two new *bla*_CMY-2-type_ genes located within a *C. freundii*-derived fragment. Considering that CMY-type β-lactamases, detected in *E. coli*, are derived from the *C. freundii* chromosomal AmpC [1] and that chromosome-derived genes are usually mobilized by MGE [24,25], the presence of three resistance regions with diverse resistance determinants and MGE in this study, suggests the dynamics of bacteria in the transference of antibiotic resistance. In addition, they might also trigger the future emergence and spread of these resistant determinants both at a chromosomal or/and plasmid level.

## Availability of supporting data

The data set supporting the results of this article is included within the article.

## Abbreviations

PMAβ: Plasmid-mediated AmpC-β-lactamase
SFM: French Society of Microbiology
MDR: Multidrug-resistance
MGE: Mobile genetic elements
ESAC: Extended-spectrum AmpC cephalosporinases
ESBL: Extended-spectrum β-lactamase
